# Supplementary material for: A Critical View of the Application of the APEX Software (Aqueous Photochemistry of Environmentally-Occurring Xenobiotics) to Predict Photoreaction Kinetics in Surface Freshwaters
Source: Molecules. 2019 Dec 18;25(1):9. doi: 10.3390/molecules25010009 (PMC7017383; doi:10.3390/molecules25010009)
Supplement: Supplementary file 1 [file molecules-25-00009-s001.zip › 3.2.4_gcc-4.4.0.zip!/3.2.4_gcc-4.4.0/3.2.4_gcc-4.4.0/doc/octave/html/interpreter/Miscellaneous-Functions.html]

Miscellaneous Functions - Untitled


Previous: Polynomial Interpolation,
Up: Polynomial Manipulations


---

### 27.6 Miscellaneous Functions

— Function File: **poly** (a)  
> If a is a square N-by-N matrix, `poly (`a`)`
> is the row vector of the coefficients of `det (z * eye (N) - a)`,
> the characteristic polynomial of a. As an example we can use
> this to find the eigenvalues of a as the roots of `poly (`a`)`.
>
> ```
>           roots(poly(eye(3)))
>           => 1.00000 + 0.00000i
>           => 1.00000 - 0.00000i
>           => 1.00000 + 0.00000i
> ```
>
> In real-life examples you should, however, use the `eig` function
> for computing eigenvalues.
>
> If x is a vector, `poly (`x`)` is a vector of coefficients
> of the polynomial whose roots are the elements of x. That is,
> of c is a polynomial, then the elements of
> d `= roots (poly (`c`))` are contained in c.
> The vectors c and d are, however, not equal due to sorting
> and numerical errors.
>
>
>
> **See also:** eig, roots.

— Function File: **polyout** (c, x)  
> Write formatted polynomial
>
> ```
>              c(x) = c(1) * x^n + ... + c(n) x + c(n+1)
> ```
>
> and return it as a string or write it to the screen (if
> nargout is zero).
> x defaults to the string `"s"`.
>
>
>
> **See also:** polyval, polyvalm, poly, roots, conv, deconv, residue, filter, polyderiv, polyinteg.

— Function File: **polyreduce** (c)  
> Reduces a polynomial coefficient vector to a minimum number of terms by
> stripping off any leading zeros.
>
>
>
> **See also:** poly, roots, conv, deconv, residue, filter, polyval, polyvalm, polyderiv, polyinteg.
